# Techniques for Success in Nipple-Sparing Mastectomy and Immediate Reconstruction

**DOI:** 10.3390/jcm14124363

**Published:** 2025-06-19

**Authors:** Jenn J. Park, Carter J. Boyd, Kshipra Hemal, Thomas J. Sorenson, Chris Amro, Nicholas A. Vernice, Alexis C. Lakatta, Oriana Cohen, Mihye Choi, Nolan S. Karp

**Affiliations:** Hansjorg Wyss Department of Plastic Surgery, New York University Langone Health, 550 1st Ave, New York, NY 10016, USA; jenn.park@nyulangone.org (J.J.P.); carter.boyd@nyulangone.org (C.J.B.); kshipra.hemal@nyulangone.org (K.H.); thomas.sorenson@nyulangone.org (T.J.S.); chris.amro@nyulangone.org (C.A.); nicholas.vernice@nyulangone.org (N.A.V.); alexis.lakatta@nyulangone.org (A.C.L.); oriana.cohen@nyulangone.org (O.C.); mihye.choi@nyulangone.org (M.C.)

**Keywords:** nipple-sparing mastectomy, breast reconstruction, direct-to-implant, implant, prepectoral, subpectoral

## Abstract

**Background:** Nipple-sparing mastectomy (NSM), given demonstrated oncologic safety, is widely used for both therapeutic and prophylactic mastectomy. The popularity of NSM has spurred advancements by breast and plastic surgeons, liberalizing the indications for NSM and improving patient and aesthetic reconstructive outcomes. This review explores these developments and establishes up-to-date surgical tenets for successful NSM and reconstruction. **Methods:** A comprehensive literature review was conducted using the PubMed, Google Scholar, and Cochrane Library databases, focusing on peer-reviewed studies published up to 2024. Articles were selected based on relevance, quantity, and documentation of clinical outcomes and patient satisfaction. **Results:** NSM is utilized frequently for both invasive breast cancers and prophylactic mastectomy, with expanded criteria for candidacy by breast surgeons. Staged procedures such as adjunct reduction, mastopexy, or nipple delay allow patients with larger or ptotic breasts to undergo NSM with comparable outcomes. Long-term outcome studies have identified important risk factors for complications, including smoking history, higher mastectomy weight, certain medical comorbidities, and suboptimal mastectomy flaps. Evolutions in reconstructive decision making in direct-to-implant and staged tissue expander placement have improved aesthetic results while accounting for poor mastectomy flap quality or adjuvant therapy. Long-term outcomes show NSM remains safe and has comparable rates of local recurrence. Patient-reported outcomes demonstrate satisfaction with NSM, especially in sexual and psychological wellbeing metrics. **Conclusions:** NSM has been demonstrated to be safe in long-term oncologic outcomes. Its widespread popularity over the past ten years has helped identify methods to improve upon surgical and aesthetic outcomes, including decision-making in reconstruction; considerations for challenging patient-related characteristics such as macromastia, ptosis, and NAC asymmetry; and novel advances in areas such as neurotization.

## 1. Introduction

Nipple-sparing mastectomy (NSM) has increased in popularity as both a surgical treatment for invasive breast cancer and a technique for prophylactic mastectomy in high-risk patients [1]. Studies have demonstrated that NSM is oncologically safe, with acceptable long-term recurrence outcomes and comparable postoperative complication rates compared to skin-sparing mastectomy (SSM) [2,3,4]. Outcome studies suggest NSM leads to higher patient satisfaction and improved aesthetic results, further spurring the popularity of NSM [5,6,7].

With this widespread adoption of NSM, both breast surgeons and plastic surgeons alike have progressed in their techniques and utilization of NSM. A better understanding of the risk factors and outcomes in NSM has led to significant evolution in the indications, surgical techniques, and aesthetic and reconstructive principles used in NSM. As many centers have overcome the initial technical learning curve and are reflecting on outcomes over the past decade, a new standard dogma is replacing the pearls of NSM developed in the early 2010s.

Immediate reconstruction with either direct-to-implant (DTI) or staged reconstruction with tissue expanders (TEs) provide a range of reconstructive options for patients including “breast in a day” [8]. The decision tree for the timing of reconstruction and the plane of prosthesis placement is dependent on both preoperative patient factors, intraoperative assessment of mastectomy flaps, and requirement for adjuvant therapies. Although risk assessment models have been published in the literature, this decision-making still relies on the clinical gestalt of the operating plastic surgeon. Use of adjunct techniques, including biologic mesh placement, nipple delay, or reduction procedures, may also affect decision making and aesthetic outcomes.

In this review, we aim to provide an overview of the current practices for NSM and implant-based reconstruction as well as provide our clinical insights as informed by experience performing NSM at a high-volume center over the past decade. We will review indications for NSM and immediate reconstruction, discuss surgical techniques for implant-based reconstruction with DTI and staged TE reconstruction, and summarize long-term safety outcomes.

## 2. Materials and Methods

A comprehensive literature review was conducted using multiple databases, including PubMed, Google Scholar, and Cochrane Library Databases. The search focused on articles published up to 2024, with heavy emphasis on outcomes published in the past decade since 2014. Keywords included a combination of the following: “nipple sparing mastectomy,” “surgical outcomes,” “oncologic outcomes,” “implant reconstruction,” “tissue expander,” “immediate reconstruction,” “delayed reconstruction,” “subpectoral,” “prepectoral,” “dual-plane,” “mastectomy flap,” “incision,” “neurotization,” “mesh,” “synthetic mesh,” “biologic mesh,” “non-synthetic mesh,” “aesthetic,” “complications,” and “patient satisfaction.”

Articles were selected based on reports of relevant clinical outcomes or surgical techniques in immediate implant-based reconstruction for NSM. Topics of interest included indications for NSM; surgical techniques in reconstruction, including incision placement, DTI and staged TE reconstruction; adjunct techniques such as use of mesh, concurrent reduction procedures, and neurotization; aesthetic outcomes; and surgical complications. All articles were reviewed independently by two authors (JP and CB) to ensure thoroughness and accuracy in selection.

### 2.1. Preoperative Evaluation and Patient Selection

Patient selection is crucial for success in NSM from both an oncologic and aesthetic perspective. Past medical and surgical history, body mass index (BMI), and smoking status should be carefully assessed preoperatively. A smoking history of more than 10 pack-years or less than 5 years since quitting has been shown to have increased rates of nipple–areolar complex (NAC) necrosis [9]. Overall breast symmetry, breast footprint, and any chest wall deformities should be noted, in addition to the desired breast size and plan for intervention in the unaffected breast. Any scars from prior breast surgery, including reduction, mastopexy, or lumpectomy, should be noted and taken into consideration for future surgical planning. Periareolar scars are especially important to note as recent incisions may increase the risk for NAC complications.

While the rise in popularity of NSM was partly attributed to the increasing popularity of risk-reducing or prophylactic mastectomy, studies show the majority of NSMs are still performed for oncologic indications [10]. Initial indications for NSM included small tumor size (less than 3 cm), tumor distance of at least 2 cm from the nipple, negative axillary nodes, no skin involvement of breast disease, and a negative intraoperative frozen section from the retroareolar region [11]. Patients with large or ptotic breasts were thought to be ineligible for NSM based on the difficulty of managing excess skin during the resection and limiting the final aesthetic result of reconstruction [11].

In recent years, increasing use of NSM has led to liberalization of indication criteria by both breast surgeons and plastic surgeons from an oncologic and aesthetic standpoint (Table 1). As breast surgeons have gained more experience with NSM, the importance of tumor size in considering candidacy for NSM has decreased, with some breast surgeons suggesting that NSM can be performed for any tumor size that does not involve the skin or NAC [12,13]. For large tumors that historically would have been challenging to resect using NSM, teams are opting to proceed with neoadjuvant therapy in hopes of reducing the tumor size and downstaging the tumor with the aim of improving the feasibility of NSM [12,14,15].

The tumor-to-nipple distance is an important consideration for candidacy in NSM. Traditionally, a distance greater than 2 cm was recommended for pursuing NSM. More recently, studies have suggested that this safe distance may be reduced, as the risk of locoregional recurrence may be comparable in tumor-to-nipple distances of 1–2 cm. Oncologic outcome studies have shown that a distance from the nipple of less than 2 cm does not have a significantly higher rate of locoregional recurrence [16,17]. Our institutional data suggests that a tumor-to-nipple distance of less than 1 cm is a significant risk factor for local recurrence [18]. Imaging-based outcome studies have suggested a minimum tumor-to-nipple distance of 1–2 cm [19]. Notably, a shorter tumor-to-nipple distance may increase risk for NAC-related complications [20]. The management of positive nipple margins or pathologic retroareolar biopsy findings such as atypia is at the discretion of the breast surgeon, with options including further surgical resection ranging from subareolar shave biopsy to nipple or areolar excision, planned future radiotherapy, or observation. This management may vary widely based on the preferences of the breast surgeon and individual cases [21,22].

Adjunctive surgical techniques for management of large and/or ptotic breasts in NSM has evolved significantly over the past decade. Techniques such as staged operative intervention to incorporate nipple delays, breast reduction and mastopexy techniques, and nipple grafting have allowed breast groups to expand indications to include patients who previously would not have been ideal candidates for NSM. Several groups have reported the use of mastopexy and/or breast reduction prior to completion mastectomy and reconstruction or incorporating simultaneous mastopexy at time of NSM using a reduction pattern incision [23,24,25,26,27,28,29,30,31]. Some groups have reported the use of free nipple grafting to aid with nipple positioning in ptotic patients, though standard reduction mammaplasty techniques may have improved aesthetic outcomes compared to nipple grafting [32,33].

Some surgeons aim to prevent NAC-related complications by incorporating a “nipple delay,” in which blood supply to the NAC is partially compromised prior to formal NSM. This procedure works especially well in patients undergoing prophylactic mastectomy, where time to mastectomy does not have oncologic consequences. In patients undergoing therapeutic mastectomy, the initial nipple delay stage may also include a lumpectomy to avoid delay of oncologic treatment. Nipple delay has shown successful outcomes with complication rates comparable to those of traditional NSM candidates [34,35].

The patient population with macromastia and ptotic breasts is more likely to have higher BMIs and higher mastectomy weights, factors known to increase risk of postoperative complication rates. Compared to smaller-breasted patients, however, rates of skin and nipple necrosis-related complications appear to be comparable with appropriate use of staged reconstruction [23,24,35]. For therapeutic mastectomies, incorporating staged reconstruction results in a delay to completion mastectomy by 3–4 months based on reported protocols [23,25]. Given the possible need for neoadjuvant therapies, the delay between the initial oncoplastic reduction and completion mastectomy may be inadvertently prolonged to accommodate systemic therapy. To avoid the need for two-stage reconstruction, few groups have reported a single-stage mastopexy and NSM technique, relying on de-epithelialization and adipodermal flaps to maintain NAC perfusion [29,30].

An additional subset of patients presenting for NSM and reconstruction that may be at greater risk for reconstructive complications are those with prior lumpectomy/radiation treatment. Patients with prior radiation therapy to the breast are shown to be associated with an increased complication rate with reconstruction, including higher nipple loss and reconstruction failure rates [21,36]. Furthermore, in patients known to require future radiation therapy, surgeons may tend to be more hesitant in offering NSM, given these known risks, as a combined history of previous radiation therapy and adjuvant radiation therapy has been found to increase complication rates and rates of surgical revision [36]. This complication rate may be compounded with the presence of other risk factors such as smoking and higher mastectomy weight, with complication rates reported as high as 66% [36].

Synthesizing factors important in determining candidacy for NSM, studies have found that patients who ultimately undergo NSM compared to SSM tend to be younger, with a lower BMI, lower mastectomy weights, and lower stage of disease [2]. Given the variabilities in practice that can be seen, success in reconstruction is highly dependent on the experience of the breast surgeon and a trusted working relationship between the breast surgery and plastic surgery teams [12].

**Figure 1:**
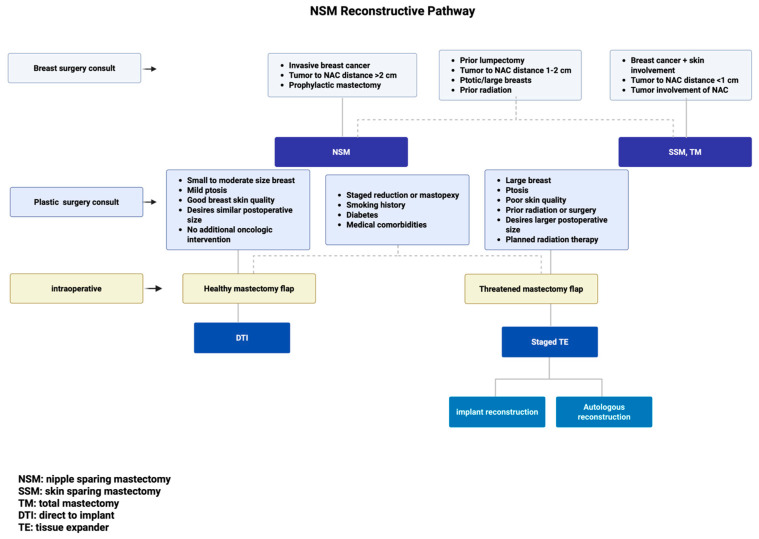
NSM Reconstructive Pathway.

In our practice, once patients are deemed to be oncologic candidates for NSM by the breast surgery team, reconstructive candidacy is reviewed based on the patient exam and preferences (Table 2, Figure 1). For patients with small to moderate-sized breasts and mild ptosis (sternal notch-to-nipple distance < 27 cm) with good breast skin quality and minimal laxity (pinch test > 2 cm, snap-back in each breast quadrant) who desire a similar postoperative breast size, immediate one-stage implant-based reconstruction is a possibility [37]. For patients interested in a substantially larger breast size following reconstruction, or for those who require neoadjuvant chemotherapy or RT, staged reconstruction with TE(s) is advised. For patients desiring autologous reconstruction, candidacy and donor site options are discussed, with options for immediate autologous reconstruction or staged autologous reconstruction with initial TE placement. For patients with significant macromastia or ptosis, the increased risk for ischemic complications and poor aesthetic outcomes, including NAC malposition, is discussed, and patients are recommended to undergo NSM after staged reduction/mastopexy or opt for SSM. All patients are counseled on intra-operative assessment of mastectomy flaps or subareolar biopsy that may change the operative plan. These changes include converting to two-staged reconstruction or delaying reconstruction until optimal healing of mastectomy flaps. In patients with unilateral disease who are not pursuing contralateral prophylactic mastectomy, symmetrizing procedures such as mastopexy or reduction are discussed based on patient preferences for intervention. The contralateral symmetrizing procedure may be addressed concurrently if the patient undergoes immediate DTI reconstruction. If the patient is planned for staged reconstruction or will require adjunct therapies, delayed timing for the contralateral symmetrizing procedure is discussed. Patients are counseled that there may be aesthetic and sensory differences between the reconstructed breast and the native breast even after the symmetrizing procedure, especially if there is a plan for unilateral radiation therapy. The management of the contralateral nipple, if the NAC must be removed during mastectomy, also needs to be discussed.

### 2.2. Surgical Techniques

#### 2.2.1. Incision Placement

Incision placement in NSM has been shown to affect the ischemic complication rate and should be determined by collaborative discussion with the breast surgeon. Incision options include the inframammary fold (IMF), lateral radial, vertical radial, and periareolar incisions. Success of NSM with each of these incisions is highly dependent on the breast surgeon’s experience—for example, one team has reported preference of a lateral incision for inexperienced breast surgeons to maximize the chances of successful blood supply to the NAC [38]. Incision options may also be limited based on the size and location of the tumor; among a national breast surgeon consortium, a majority felt that IMF incisions should not be used for tumors in the upper inner quadrant in large breasts [12].

Studies show the highest complication rates with the periareolar incision, with reported complication rates as high as 42.6% of patients [20]. The inframammary fold (IMF) incision has been shown to be protective against NAC complications [20,39]. Data from our institution shows that in immediate autologous reconstruction, the IMF incision may lead to higher complication rates compared to vertical and lateral radial incisions. This may be caused by the degree of retraction needed for the exposure of chest vessels [40].

In patients with significant macromastia or ptosis, simultaneous NSM and breast lift utilizing a Wise pattern reduction incision or similar variation has been described [29,30,31]. As reduction pattern incisions have been associated with increased NAC complications, these techniques maintain NAC vascularity by de-epithelializing within markings (similar to skin-only mastopexy) or by maintaining a separate de-epithelialized adipodermal flap [28]. Reported rates of NAC-related complications with these techniques range from 5 to 9% [29,30].

Groups have also experimented with transaxillary NSM and endoscopic implant reconstruction, suggesting that this approach may be useful in patients with prior breast surgery or scars that may compromise the flap or NAC perfusion [41,42,43]. With recent increasing adoption of robotic surgery, breast surgeons and plastic surgeons have begun to trial robotic NSM and reconstruction [44,45,46,47]. Though reported outcomes have demonstrated feasibility and positive aesthetic results, significant challenges in the learning curve and required resources for robotic surgery have limited widespread adoption of these techniques.

A case series of NSM in patients with prior breast surgery showed that most patients underwent mastectomy using their primary surgical incision with NAC and mastectomy flap necrosis rates of approximately 5%. There was no association between complications and the type of skin incision for the index operation or mastectomy, repeat use of the same incision, or type of the index surgery [48]. For ptotic breasts, the use of a skin reduction pattern, including modified Wise pattern incisions, has been used but is at significant risk for wound healing complications [40].

#### 2.2.2. Mastectomy Flap Quality

Mastectomy flap quality is important in any breast reconstruction but especially in NSM as the entire breast envelope and NAC is supported through the subdermal plexus and remaining subcutaneous vasculature. For this reason, there have been significant efforts to quantify breast flap thickness and its effects on both breast cancer recurrence and mastectomy flap-related complications. A recent systematic review found that breast flap thickness as measured radiologically has been reported to range from 3.8 to 23 mm, and that thickness over 5 mm may be a risk factor for cancer recurrence [49,50]. From the plastic surgeon’s perspective, a thicker mastectomy flap lowers the chances of ischemia-related complications that may threaten the reconstruction. However, breast flap thickness, effectively the amount of subcutaneous tissue above the breast capsule, is dependent on BMI and breast size and is not uniform across patients. Given that flap thickness is variable, and intraoperative measurements of thickness are difficult to obtain, intraoperative assessment of the mastectomy flap is largely dependent on the clinical gestalt of the operating plastic surgeon. This assessment may be augmented with the use of intraoperative indocyanine green (ICG) angiography, which can provide a real-time demonstration of blood flow to the mastectomy flap. However, interpretation of this inflow remains largely subjective. Studies have shown that use of intraoperative ICG is associated with lower rates of mastectomy flap-related complications; early investigations are exploring whether fill area, fill time, or fill intensity cutoffs can be identified to predict mastectomy flap necrosis [51,52,53].

In the breast surgery literature, there is significant variability in reported practice patterns for techniques to ensure mastectomy flap viability. Approximately 50% of breast surgeons reported using an intraoperative skin flap viability assessment in conjunction with the reconstructive surgeon. Some breast surgeons routinely use topical nitroglycerine either when concerned about threatened flaps or as a prophylactic measure [12]. Breast surgeons agree on the importance of gentle retraction on skin flap viability; the effects of sharp dissection versus cautery have not been well established and are often dependent on breast surgeon preference [12]. Ultimately, the mastectomy flaps, as assessed by the plastic surgeon upon conclusion of the mastectomy, will affect the decision for prepectoral or subpectoral reconstruction and DTI or staged reconstruction with TEs. For this reason, part of the success of reconstruction is heavily reliant on working with a trusted breast surgeon, and patients should be counseled extensively on all potential reconstructive options preoperatively.

#### 2.2.3. Direct to Implant Reconstruction

Patients with moderate-sized breasts with minimal ptosis who desire a similar postoperative breast size are ideal candidates for DTI reconstruction. Immediate, permanent implant reconstruction allows for fewer reconstructive procedures and shorter time to the final reconstructive result, minimizing operative visits and the psychological burden on patients [54].

Complications in DTI reconstruction include NAC or mastectomy flap necrosis, poor wound healing, seroma, or hematoma, which may lead to implant exposure and/or infection, requiring explantation. Reconstruction failure in DTI reconstruction can be devastating, as management will likely require temporary closure and healing prior to reattempting reconstruction and may require staged tissue expansion. In the literature, reported rates of implant loss range from 4 to 15% [38,50,55]. DTI reconstruction with NSM at our institution has shown a 4.4% rate of implant loss. As discussed above, older age > 50 years, implant size > 400 cc, smoking history, and prior radiation therapy are significant risk factors for complications in DTI reconstruction [54].

#### 2.2.4. Subpectoral Versus Prepectoral Reconstruction

Prepectoral and total or partial submuscular implant placement are both widely used in NSM for implant-based reconstruction. Aesthetically, prepectoral reconstruction can more easily achieve medial fullness and proper NAC positioning as the pectoralis muscle does not impede implant positioning and the prosthesis can be placed up to the sternal edge. Prepectoral reconstructions have consistently shown reduced levels of postoperative pain and improved upper extremity function compared to submuscular reconstruction, and they avoid the long-term risk of animation deformity [56,57]. Prepectoral reconstruction may also have lower rates of capsular contracture after mastectomy compared to subpectoral reconstructions [56]. However, prepectoral placement requires well-vascularized mastectomy flaps as the implant sits directly under the skin envelope and is vulnerable to exposure or infection if the skin envelope is compromised. There is also risk of visible implant rippling, especially in thin patients, often requiring ancillary procedures including autologous fat transfer [58].

Subpectoral implant placement, whether dual-plane or total submuscular coverage, provides a layer of vascularized muscle over the prosthetic, thereby providing the implant with an additional layer of protection in the event of incisional dehiscence or mastectomy flap necrosis [59]. Achieving natural breast contour and NAC positioning may be more difficult in subpectoral reconstructions, as the submuscular pocket that is developed must closely match the overlying skin envelope. There is risk of long-term animation deformity and upper extremity pain or weakness with subpectoral implant placement [56]. Breast size, ptosis, and contralateral symmetrizing procedures also factor into this decision. In unilateral reconstruction, it may be difficult to match the contralateral breast in larger or ptotic breasts with subpectoral reconstruction.

Historically, practice patterns have oscillated between prepectoral and subpectoral implant placement based on trends and reported complications for both aesthetic and reconstructive use of breast implants. While there has been a resurgence of prepectoral reconstruction, the reported literature in the last 10 years consistently includes both prepectoral and subpectoral techniques, and no surgical group is able to rely on one plane of reconstruction completely for all patients [60,61].

Given that prepectoral reconstruction relies solely on coverage of the implant by the mastectomy flap, there is increased concern for the risk of infection and potential reconstruction failure with prepectoral placement. Reported outcomes in the literature have been inconclusive regarding the effect of plane placement on complication rates in NSM. Some studies have suggested there are no differences in complication rates for the subpectoral and prepectoral approach [38,62,63]. Other studies have suggested that prepectoral reconstructions have higher rates of surgical site infection, cellulitis, seroma, and expander and implant loss, as well as a total explantation rate of up to 13–22% [60,61]. One study suggested that prepectoral reconstructions present with infection earlier than subpectoral reconstruction (95 vs. 160 days) and are more prone to infection with Gram-negative organisms [60].

Comparing outcomes in prepectoral and subpectoral implant placement remain challenging due to the inherent patient selection bias between groups. Patients with better-quality mastectomy flaps are more likely to be chosen for prepectoral reconstruction, which may be due to both patient-related factors or intraoperative assessment. For example, one systematic review meta-analysis found that prepectoral reconstructions had lower rates of mastectomy flap necrosis compared to subpectoral reconstructions [64]. However, subpectoral reconstruction patients were also found to have a higher incidence of tobacco use, thus limiting direct comparison between groups.

Ultimately, the decision for prepectoral versus subpectoral reconstruction is made at two points in the reconstructive timeline: (1) preoperatively, based on the assessment of native soft tissue envelope/pinch test, the degree of ptosis and required symmetry with the contralateral breast, and patient lifestyle factors such as weightlifting or the desire to maintain maximum strength of pectoral muscles; and (2) intraoperatively, based on the assessment of mastectomy flap viability. Patients should be appropriately counseled that the reconstructive surgeon may have to adapt the plan based on intraoperative findings.

#### 2.2.5. Use of Soft Tissue Support

Soft tissue support with mesh or scaffolds is a frequently used adjunct, especially in prepectoral reconstruction, where it can be used to support the implant or TE within the mastectomy pocket. A variety of soft tissue support modalities have been described for use, including acellular dermal matrix (ADM), vicryl mesh, polydioxanone (Durasorb), titanium coated polypropylene (TiLOOP), glycolide and lactide (TIGR), and poly-4-hydroxybutyrate (P4HB) [65]. The addition of an avascular tissue layer, though potentially helpful for support and positioning, may trigger unwanted inflammatory reactions or act as a potential nidus for infection. There have been conflicting single-institution reports and systematic reviews on the effect of mesh use on both negative complications such as hematoma, seroma, infection, or explantation rates, as well as potential benefits such as decreased capsular contracture and reduced visible rippling [38,66]. However, many outcome studies do not consistently report on the use of soft tissue support, and soft tissue support is not consistently used in all prepectoral reconstructions [66,67]. Comparative data between types of soft tissue support is limited. Furthermore, no soft tissue support mesh or scaffold is FDA-approved for use in breast reconstruction and is thus used off-label [68]. Ultimately, the use of soft tissue support is often surgeon-specific, with limited data on the superiority of one product versus another or firm guidelines indicating its use.

Although the most used soft tissue support reported in the literature has historically been ADM, other modalities have been utilized over the past two decades. Like many surgeons, our practice has evolved across time with the aim of finding the most effective soft tissue support while minimizing complications. When it was first introduced, we utilized ADM, which was effective in assisting with prosthesis support and inferior pole coverage in dual-plane implant placement. However, higher rates of infection and implant explantation in our ADM cohort led us to become more selective about ADM use [69]. We trialed alternative soft tissue support materials including vicryl mesh and polydioxanone mesh primarily for prepectoral or dual-plane reconstructions, with pooled analysis supporting our thought that soft tissue support helps reduce complication rates in prepectoral reconstruction [70,71]. More recently, we have been shifting towards the use of poly-4-hydroxybutarate (PH4B, GalaFLEX) to aid in the reduction in dead space and micro-shifting of the implant within the prepectoral pocket [72,73]. The utilization of PH4B scaffolds in breast reconstruction is still in its early phases and long-term data is needed to determine the risks and benefits of use [65,74,75].

#### 2.2.6. Neurotization

Sensory restoration in breast reconstruction has remained challenging without consistent outcomes. Although overlying breast skin may regain some degree of sensation over time after mastectomy, the timing and degree of sensory return remain highly variable [76]. Neurotization in autologous reconstruction has been consistently explored with direct nerve coaptation or combined use of conduits and nerve allografts; however, neurotization in implant-based reconstruction has not been widely studied [77,78]. There are currently preliminary investigations preserving the anterior branch of the lateral fourth intercostal nerve during mastectomy and using interposition allografts to reinnervate the NAC [79,80]. However, long-term outcomes are needed to determine whether this intervention is successful in restoring clinically meaningful sensation.

#### 2.2.7. Staged Reconstruction with Tissue Expanders

Reconstruction may be performed in a staged fashion using tissue expanders (TEs) followed by implant placement or autologous reconstruction. Tissue expanders are helpful in maintaining the pocket for reconstruction before definitive reconstruction and are often used in patients requiring adjuvant radiation therapy followed by staged or delayed reconstruction, or in those with threatened mastectomy flaps intraoperatively.

In comparison to DTI reconstruction, TE reconstruction does not initially place volume-induced stress on the mastectomy flaps. Theoretically, this may lead to fewer ischemic complications in the immediate postoperative period such as nipple necrosis and mastectomy flap necrosis, which has been shown in our institutional data [81]. However, there is poor consensus in the reported literature, likely because patients undergoing TE reconstruction are more likely to have suboptimal mastectomy flaps, and the process of expansion increases physical stress on overlying skin and presents an opportunity for infection. In this vein, some centers have found that TE reconstruction in NSM has higher rates of nipple necrosis, infection, and explantation compared to DTI, regardless of the plane of prosthesis placement [38]. Patient characteristics that predispose patients to requiring TE reconstruction, such as age, large breast size (larger than C cup), previous radiation, repeat implant placement, and delayed reconstruction, are also independent risk factors for postoperative infection, making it difficult to isolate the cause for the observed complication rates [82,83,84].

The decision for TE placement in the prepectoral or subpectoral plane follows the guidelines for DTI reconstruction discussed previously. Prepectoral TE reconstruction may be more susceptible to infection or mastectomy flap necrosis, with rates reported to be as high as 27% [85,86]. Prepectoral reconstruction has been shown to have shorter time to reconstruction, though this may be a result of patient selection as patients chosen for prepectoral TE reconstruction were also less likely to receive adjuvant radiation [85].

In staged reconstruction, it is important to note that the symmetry achieved at the TE stage is indicative of the final aesthetic result. Any nipple asymmetry noted at the TE stage is likely predictive of final nipple positioning, though symmetry may also be altered by radiation [87,88]. Interestingly, one study showed that compared to TE reconstruction, DTI may have increased nipple asymmetry, especially in prepectoral reconstructions [89].

Staged reconstruction is frequently used in patients requiring adjuvant radiation out of an abundance of caution for the known risks of radiation therapy on the skin envelope. However, reported data suggests that complication rates, though significantly higher compared to patients who do not undergo radiation, may be comparable between radiation with TE and implants. Several studies have shown that complication rates are comparable when undergoing radiation therapy before or after TE–implant exchange [90,91,92,93]. Though few surgical teams opt for DTI reconstruction in patients requiring radiation therapy, one study found that patients receiving post-mastectomy radiation therapy with TE reconstruction had higher rates of reconstruction failure than those who received DTI reconstruction, while those patients undergoing reconstruction with either modality in the absence of radiation had no differences in complication rates [55]. This finding may be attributed to the thickness/perfusion of the skin envelope, in which patients undergoing DTI reconstruction are selected with well-perfused mastectomy flaps, while skin flaps in TE reconstruction are exposed to additional pressure and insult through the TE expansion process. However, the high rates of prosthesis exposure and reconstruction failure in this group make TE reconstruction the safer choice in patients requiring post-mastectomy radiotherapy.

### 2.3. Clinical Outcomes

#### 2.3.1. Oncologic

At its inception, concerns regarding NSM surrounded its ability to provide comparable oncologic outcomes to other techniques. Now approaching two decades of experience, there is sufficient follow-up data to determine its comparative efficacy. NSM is equally as safe as skin-sparing mastectomy (SSM), with no difference in 5-year survival and mortality rates [94]. Reported local recurrence rates are acceptable and range from 2.5 to 3.1% at up to 10 years of follow-up [3,39,95]. Given its established safety, there has been a significant increase in NSMs performed for invasive breast cancers, with reported 77–85% of therapeutic NSMs performed for invasive tumors [1,10,17].

Currently, no demographic, operative, or tumor subtype-specific risk factors have been consistently identified as independent risk factors for recurrence; however, the overall low incidence of local recurrence likely precludes the identification of such trends. More broadly, pathologic findings including lymphovascular invasion, multifocality, and multicentricity of the breast cancer are significant risk factors for breast cancer recurrence, though this finding is not specific to NSM [96]. Low recurrence rates are maintained by prudent safeguards, such as appropriate conversion to SSM when indicated; approximately 4–10% of patients require nipple excision intraoperatively due to positive margins [1,2,3]. Close collaboration and established protocols between the breast and plastic surgery teams, in addition to thorough preoperative planning and patient counseling, are necessary to address intraoperative changes in the surgical plan for resection and reconstruction.

As NSM is typically indicated for both breast cancer and prophylactic mastectomy, the cohort of NSM patients tend to be younger, with private insurance, lower BMI, lighter breasts, and less likely to require axillary dissection or neoadjuvant therapy compared to patients undergoing SSM, which usually represents a cohort with more advanced disease [2,97]. When controlling for these factors, NSM may have a higher complication rate compared to patients of similar demographics undergoing SSM, due to the frequency of nipple ischemia-related complications [2]. Rates of nipple ischemia-related complications requiring reoperation have been reported to be between 5 and 16% [1,2,23,98].

Prophylactic NSM has identified cancer or significant atypia in 4–10% of specimens, with the most common pathologies being ductal carcinoma in situ (DCIS) and atypical lobular hyperplasia [4]. Long-term data assessing cancer occurrence rates in patients undergoing prophylactic NSM demonstrates extremely low incidences of breast cancer across a time period of more than a decade [4].

#### 2.3.2. Aesthetic

Aesthetic results in NSM are highly dependent on nipple positioning and symmetry [99]. Patients must be counseled preoperatively on any existing nipple asymmetry or chest wall deformity that may affect reconstructive results. Patients may have differing opinions on ideal NAC position and size, which are important to take into consideration when planning reconstruction. Intraoperatively, NAC positioning may be secured to the underlying muscle or mesh to help control the final aesthetic result [37,81]. This may be easier to achieve in prepectoral reconstruction as the skin envelope is draped directly over the implant.

As discussed previously, nipple position at initial reconstruction, whether TE or DTI placement, is likely to persist over time. Asymmetry in implant-based reconstruction is typically due to the draping of the NAC and skin envelope over the prosthesis, which is more difficult to control with macromastia or ptosis despite adequate positioning of the implant on the chest wall. Late asymmetries may develop secondary to capsular contracture, distorting breast shape and nipple position. Additionally, radiation, lateral radial mastectomy incisions, or autologous reconstruction have been found to be predictive for NAC asymmetry requiring subsequent intervention [81]. Our institutional data has shown that approximately 7% of patients elected to undergo secondary surgery for NAC repositioning [81].

Asymmetry may be addressed via crescentic periareolar excision, capsule modification, or direct skin excision based on the source of NAC asymmetry. Careful consideration of the blood supply to the NAC must be given during revision surgery, and the subdermal plexus of the prior mastectomy flaps should be conserved as much as possible to avoid secondary wound healing complications. Finally, aesthetic complaints with the NAC itself, including size, pigmentation, or deformity secondary to NAC necrosis, may be addressed with local excision, areolar skin flaps, or tattooing.

#### 2.3.3. Patient Satisfaction

There has been a significant effort to quantify patient satisfaction outcomes associated with NSM. A main challenge is that the BREAST-Q was created before the rise in popularity of NSM and does not include NAC-specific questions. Multiple studies using the BREAST-Q lack consistency in identifying the specific benefits of NSM, though the majority of results skew towards higher psychosocial and sexual wellbeing scores in NSM compared to SSM [6,100,101]. Conversely, other studies have shown no difference in the BREAST-Q scores of NSM patients compared to SSM patients, especially regarding satisfaction with breasts [5,6,97]. One study suggests that the benefits achieved by NSM may attenuate with time, with greatest impact seen within the first five years postoperatively and diminishing 6–10 years post reconstruction [7].

Unfortunately, studies utilizing the BREAST-Q fail to specifically address NAC-related factors. BREAST-Q results are also known to be affected by radiation and BMI, regardless of the type of mastectomy and reconstruction [7]. There have been efforts to develop a nipple-specific scale for the BREAST-Q to better address patient satisfaction outcomes in NSM, but currently there is a lack of widespread adoption and follow-up of these patient-reported outcome metrics [102]. Establishing a validated, easily accessible nipple-specific scale will allow better characterization of the benefits of NSM, including parsing out how sensation and erogenous function (or lack thereof) of the NAC affect patient-reported outcomes. This will also help guide future directions in fields such as neurotization.

#### 2.3.4. Limitations

Although this review aims to highlight current trends and recent advances in NSM and implant-based breast reconstruction, our study is not without limitations. Published data in the literature skews towards high-volume, resource-heavy academic centers and may not accurately reflect the practice patterns in community hospitals or rural settings. Furthermore, the pathway of decision-making to undergo NSM and reconstruction is influenced by the clinical gestalt of the operating breast surgeon and plastic surgeon, patient population, and available resources. Clinical decision making for the plastic surgeon, in addition to considerations discussed in this review, is dependent on the need and timing of adjunct therapies for breast cancer, which are beyond the scope of this study and difficult to account for. These nuances may not be fully captured or described in the literature.

## 3. Conclusions

NSM has been shown to be a safe, widely utilized option for patients with both therapeutic and prophylactic mastectomies. The increasing popularity of this procedure within the past decade has helped identify the ideal candidate for NSM as well as risk factors for reconstruction failure. As breast and plastic surgeons have become more experienced with NSM and reconstruction, there has been an evolution of indications for NSM, modifications of techniques to increase candidacy for NSM, and new innovations to maximize surgical and aesthetic outcomes. Future work in this field will help define recommendations for subsequent advances in the field, including NAC neurotization and patient reported outcomes.

## Figures and Tables

**Table 1:** Indications and contraindications for NSM.

Indications	Contraindications	Special Consideration
Invasive breast cancer (including large size > 3 cm)	Breast cancer with skin involvement	Prior lumpectomy
Tumor > 2 cm away from NAC	Tumor < 1 cm away from NAC or with direct NAC involvement	Tumor 1–2 cm from NAC
Prophylactic mastectomy		Ptotic and/or large breasts
		Prior radiation therapy

**Table 2:** Considerations for DTI or TE staged reconstruction.

DTI	TE Staged Reconstruction	Special Consideration
Small to moderate-size breast (A, B, C cup)	Large breast (large C cup, D cup, or greater)	Staged reduction or mastopexy procedure
Mild ptosis (no ptosis, grade 1 ptosis, pseudoptosis, SN-N < 27 cm)	Ptosis (grade 2, 3 ptosis)	
Good breast skin quality, minimal laxity (skin pinch test > 2 cm, skin stretch in each breast quadrant)	Poor skin quality, history of prior radiation or surgery	Smoking history, diabetes, medical comorbidities
Desires similar postoperative size	Desires larger postoperative size	
Healthy mastectomy flaps	Intraoperative assessment with threatened mastectomy flaps	
No further oncologic intervention	Planned radiation therapy	
